# Socioeconomic, intrapersonal and food environmental correlates of unhealthy snack consumption in school-going adolescents in Mumbai

**DOI:** 10.1186/s12889-022-13449-6

**Published:** 2022-06-06

**Authors:** Panchali Moitra, Jagmeet Madan

**Affiliations:** Department of Food, Nutrition & Dietetics, Sir Vithaldas Thackersey College of Home Science (Autonomous), SNDT Women’s University, Santacruz West, Mumbai, 400049 Maharashtra India

**Keywords:** Adolescent snacking, Unhealthy snack consumption, Food environment, Snacking habits of adolescents, Indian adolescents, Socioeconomic status, Factors of snack consumption, Snacking behaviors of adolescents in India

## Abstract

**Background:**

Unhealthy snacking habits in adolescents are typically triggered by obesogenic food environments and possibly perpetuated through interactions between socio-environmental factors and personal perceptions, attitudes, and motivations to change eating behaviors. This study attempted to address the knowledge gap regarding the association of intrapersonal, socioeconomic, and food environmental characteristics with unhealthy snack consumption in Indian adolescents, presenting several targets for effective interventions.

**Methods:**

A representative sample of 10–12 years old adolescents (n 712) completed a cross-sectional survey including eating habits, characteristics of school and home food environments, and perceptions related to affordability, convenience, and barriers within the food environments. The frequency of unhealthy snack consumption was assessed using a validated qualitative food frequency questionnaire. Multivariate regression analyses determined the associated factors of unhealthy snack consumption in adolescents attending private and public schools.

**Results:**

The mean age of adolescents was 10.9 (1.1) years, 48.2% were girls and 53.9% attended private schools. The most frequently consumed unhealthy snacks were biscuits/ cookies (5.2d/wk) followed by wafers (3.4d/wk) and Indian fried snacks- samosa/ vada pav (2.8 d/wk). Among the public-school adolescents, the odds of unhealthy snack consumption were 0.89 times lower when meals were had frequently at the dinner table and 4.97 times higher when the perceived barriers related to the affordability of healthy snacks were greater. Maternal education (OR 0.78, 95% CI 0.66–0.82, *p* < 0.001), family income (OR 2.15, 95% CI 1.98–2.32, *p* < 0.001), availability of unhealthy snacks (OR 2.98, 95% CI 1.36–3.46, *p* < 0.001) and fruits (OR 0.57, 95% CI 0.49–0.69, *p* < 0.001) at home, having evening meals together (OR 0.71, 95% CI 0.63–0.81, p 0.031), and perceived parental control during mealtimes (OR 0.67, 95% CI 0.62–0.72, *p* < 0.001) were associated with unhealthy snack consumption in adolescents attending private schools.

**Conclusions:**

The results highlighted a pervasiveness of unhealthy snacks in adolescents’ food environments. Improving provisions and affordability of fruits and healthy snacks at schools, encouraging family mealtimes, and limiting the availability of unhealthy snacks at home whilst addressing the perceived barriers within food environments, and inculcating self-efficacy skills can improve snacking habits in Indian adolescents, irrespective of socioeconomic backgrounds.

**Supplementary Information:**

The online version contains supplementary material available at 10.1186/s12889-022-13449-6.

## Introduction

Early adolescence is a nutritionally vulnerable period of life that is marked by an increase in personal autonomy over food choices and a greater influence of the socio-environmental factors on eating behaviors as compared to childhood [1–3]. Evidence suggests that unhealthy eating habits such as increased consumption of energy-dense and nutrient-poor snacks at the expense of healthier fruits and vegetables are often established and reinforced during adolescence [4–6]. Moreover, studies have reported a ubiquitous snacking habit among adolescents [7–9] and that the foods and beverages consumed as snacks may contribute to ~ 20–25% of their daily energy intake [10, 11]. Given the well-documented understanding that poor snacking habits are typically triggered by obesogenic food environments in adolescents’ immediate settings such as schools and homes [12–14] and an expedient need to identify targets for interventions that promote healthy dietary practices during adolescence and prevent the risk of obesity and other chronic diseases later in adulthood, the determinants of unhealthy snacking behaviors in adolescents must be delineated. This attains particular importance for adolescents residing in the urban cities of rapidly globalizing economies such as India where shifts in the availability, affordability, and desirability of foods consumed are driving an unfavorable nutrition transition [15–17] and a concomitant burden of obesity amidst the persisting challenges of undernutrition continues to pose significant public health challenges [18–20].

Previous studies have identified food environmental exposures such as the sale of unhealthy foods at schools, availability, and accessibility of healthy foods at home, and familial influences such as parents’ nutrition knowledge and family dietary practices as correlates of food consumption in adolescents [21–23]. However, the majority of the relevant evidence originates from developed countries and little is known about the socio-cultural, economic, and food environmental characteristics of adolescents in India and how they come together to influence snacking habits. Furthermore, the depth and pace of the influences of food environments on snacking behaviors are likely to vary between the urban rich and urban poor due to probable inequities in access and affordability of foods [24–26]. While several studies have established a distinct socioeconomic gradient in the food consumption patterns and have reported associations between socioeconomic disadvantages and poor diet quality in adults [27], the research regarding the influence of socioeconomic status (SES) of families on adolescents’ snacking habits is limited and the socioeconomic differentials in the school and home food environments of adolescents in India have not yet been investigated.

Besides the food environmental factors of consumption patterns, there is evidence that adolescents’ attitudes and perceptions toward healthy eating can influence their snacking habits [28–31]. For instance, a recent study among 12–17 years old adolescents observed positive associations between adolescents’ autonomous motivation and dietary habits [32], and a cross-sectional study reported perceived barriers and self-efficacy as key determinants of adolescents’ eating behaviors [33]. To date, few studies have examined the school and home food environments of Indian adolescents and there are fewer studies that have explored the perceptions towards eating behaviors and food environments in adolescents in India. The few studies that were conducted were either among older adolescents (14–16 years old) attending private schools [34] or had employed qualitative study designs [35–37].

This study attempted to address the gaps in the knowledge regarding socioeconomic and food environmental exposures as correlates of unhealthy snack choices in adolescents and the critical role that the interplay of these environmental factors with adolescents’ perceived barriers within food environments, self-efficacy, and readiness to change unhealthy habits may play in moderating their snack consumption patterns. Consistent with the ecological model of health promotion, improving snacking habits of adolescents will require a comprehensive investigation of the relationship between various intrapersonal, socioeconomic, and environmental factors. Therefore, the present study was conducted to evaluate the association of demographic, socioeconomic, food environments, and perception-related factors with the consumption patterns of unhealthy snacks that are high in fat, salt, and sugar among 10–12 years old school-going adolescents in Mumbai, India.

## Methods

### Study design and setting

This cross-sectional study was conducted in six co-educational schools (three unaided private and three government-aided public schools) in Mumbai. The study sites were selected in three steps- First randomly select a zone, out of seven zones in the district of Greater Mumbai [38]. Second, select two wards at random from the selected zone (each zone comprises 3 to 5 administrative wards). Third, send invitations to twenty public schools and twenty private schools located in these selected wards to participate in the study. Of these forty schools that were sent invitations, the first three private schools and three public schools that provided permissions were selected as the study sites.

### Participants

For the selection of participants, we invited all students, attending grades 6 and 7, at each of the study sites to participate. The details of the study protocol were verbally explained in each class of the grades 6 and 7 (each grade included 2–4 classes and each class comprised approximately 30–35 students) and the information sheet and parent consent forms were sent home to obtain written, informed parental consent. Of 1086 adolescents, who were invited to participate, a total of 765 (70.4% of those invited to participate) returned signed parental consent forms, and 712 completed the questionnaire to comprise the final sample for analyses. Similar to previous studies, the type of school attended by adolescents was used as a proxy indicator of the SES [15, 39]. The study protocol was approved by Intersystem Biomedica Ethics Committee, Mumbai, India (Approval Version 02/ dated 19 February 2019). Written informed consent from parents and written informed assent were obtained from adolescents.

### Sample size estimation

The sample size was estimated to be 323 at 5% precision, 95% Confidence Interval (CI) with the assumption that 70% of urban adolescents in India practice unhealthy eating behaviors [8, 40, 41]. After taking into account a 20% non-response rate and a proportional representation of adolescents attending private and public schools, the sample size was calculated as 775.

## Measures

### Socio-demographic characteristics

Adolescents reported their age, sex, grade (grade 6 or 7), type of living arrangement (nuclear or joint), and religion (Hindu, Muslim, Christian, and others) and the parents/caregivers provided socioeconomic information- parents’ highest educational qualifications and the family’s monthly income (Table S1).

### Eating habits

Adolescents provided information about eating habits such as weekly frequencies of consuming breakfast at home, carrying lunch or snacks to school, and watching television while eating at home. Adolescents were asked ‘In the last 7 days, how many days did you perform the following activities….”. The response options, ‘never’ to ‘always, indicating < 1/week to 6–7 times/ week, were scored on a five-point scale from 0 to 4. Higher scores indicated a higher frequency of indulging in a specific eating habit.

### Consumption of unhealthy snacks

In line with the previous studies [7, 10, 42], the snacks that are high in calories, salt, and sugar content and low in nutrients were considered unhealthy. We employed a brief qualitative food frequency questionnaire (FFQ) to assess adolescents’ consumption of unhealthy snacks during the past week. The FFQ was validated as a part of our previous study to evaluate the practices related to healthy eating in 10–12 years old adolescents in Mumbai [43]. In summary, the steps involved creating a list of locally available and commonly consumed unhealthy snack items based on the review of previous studies and the results of a pilot study entailing two non-consecutive interviewer-administered 24-h diet recalls (24 h DR) in adolescents (*n* = 55) in Mumbai. This was followed by the development of a food list comprising 28 snacks that were high in fats, sugar, and salt. This FFQ draft was next pretested among adolescents (*n* = 32) who were asked to report the weekly frequencies of consuming specific foods and indicate the most appropriate portion sizes of the consumed food items in standardized household measures such as katoris (bowl or cup), spoons, and glasses. Food items that were consumed infrequently (less than once a week) and/or had lower mean daily intakes (< 10 g/d) were removed from the list. For data sorting and analysis, similar items were grouped into four broad categories- (1) Snacks high in fat/ fast foods—4 items (2) Snacks with added sugar—3 items (3) Snacks with added salt—4 items and (4) carbonated beverages—1 item. The FFQ responses were scored 0 to 4, from ‘never’ to ‘2- 3 times a day. The scores corresponding to the consumption of all snack items were aggregated to derive total unhealthy snack consumption scores, possible scores 0 to 48, with higher scores reflecting higher consumption of unhealthy snacks. The aggregate unhealthy snack consumption scores were subsequently categorized into tertiles indicating high, moderate, and low consumption. Moreover, the consumption frequencies were calculated as the number of days/ weeks by coding the response options ‘never’ as 0 d/week, ‘sometimes’ as 1.5 d/week, ‘often’ as 3.5 d/week, ‘frequently’ as 5.5 d/week, and ‘always’ as 7 d/week.

### School and home food environments of adolescents

The school and home food environment-related questions were based on a 36-item self-reported questionnaire measuring Food-Related Environments at Schools and Homes (FRESH-Q) in adolescents in India [44]. The specific items in the questionnaire were developed based on the results of focus group discussions with adolescents, parents, and teachers reported elsewhere [31] and a thorough review of other food environment-related questionnaires administered to adolescents [45–48]. In the questionnaire, the school food environment items examined the type, price, and quality of foods and beverages available for sale in the school canteens or at the nearby street vendors for the schools that did not have an on-site canteen. The adolescents were asked ‘Do you eat at the school canteen or buy foods/beverages at school?’ and ‘If yes, then how often…? Additional questions included ‘Which of the following foods and drinks are available at school to buy?’, ‘Which of the following foods and drinks do you usually buy at school?’, and ‘Why do you buy these foods? (Response options included taste, price, availability, and convenience)’.

To explore factors within home food environments, the adolescents were asked to report the weekly frequencies of availability and accessibility of unhealthy snacks at home. The participants were asked ‘In the last 7 days, how many times were the following foods available at home’ and ‘how many times were the following foods kept in easy to reach places such as countertops or kitchen cabinets? The listed food items included the same 11 unhealthy snack items and 1 carbonated beverage item as reported in FFQ. Moreover, three questions assessed the perceived parental control during mealtime. Response options were ‘strongly disagree’ to ‘strongly agree’, scored from 0 to 4, with higher scores indicating higher perceived parental control on adolescents’ consumption patterns. An additional three questions examined the family dietary habits such as weekly frequency of eating out or ordering takeaways, having at least one meal/d with family, and having meals at the dinner table.

The content validity of the questionnaire was examined by a panel of four experts including two public health nutritionists, a food environment researcher, and an academician. The principal axis method of exploratory factor analysis was used to establish the construct validity and the internal consistency was evaluated using Cronbach alpha values > 0.7 [44]. To test the reliability of the items in FRESH-Q, a sample of adolescents (*n* = 108) took a retest of the same questionnaire after two weeks and the test–retest correlation coefficient values of each item were calculated. Based on the content and construct validity measures and the internal consistency and test–retest correlation values, the items were retained or excluded in the final instrument.

### Perceptions of barriers in food environments

**Fig. 1:**
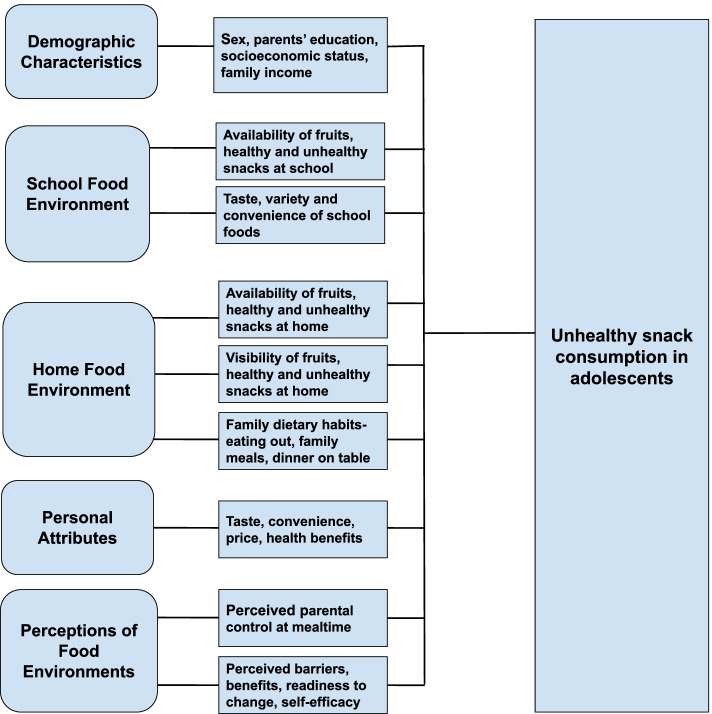
Conceptual framework illustrating the behavioral and food environmental correlates of unhealthy snack consumption in adolescents

The adolescents’ perceptions related to eating behaviors and food environments were measured using sixteen statements, based on the constructs of a widely used health behavior framework, the Health Belief Model (HBM). The items assessed adolescents’ perceived susceptibility and severity of adverse consequences of unhealthy eating behaviors, perceived barriers and benefits within food environments, and readiness to change and self–efficacy to adopt healthier dietary practices [49]. Responses to the statements were assessed on a five-point Likert scale from ‘strongly disagree to strongly agree’, with numeric scores of 0 to 4. A conceptual framework illustrating the behavioral and food environmental correlates of unhealthy snack consumption in adolescents is provided in Fig. 1.

### Pilot testing of the questionnaire

The face validity of the questionnaire including socio-demographic characteristics, eating habits, frequency of consumption of unhealthy snacks, food environments, and adolescents’ perceptions were evaluated in 34 adolescents, ages 10–12 years (20 from a public school and 14 from a private school). These participants were not included in the final data analysis. All the questions were well understood, and no revisions were considered. The field investigators having postgraduate degrees in nutrition received a full day of training to review the protocol and methods of data collection. The questionnaire was administered to adolescents during school hours in the presence of school teachers and the trained field research team. The summary of items included in the questionnaire is provided in Supplementary Material, Table S1.

### Statistical analysis

Adolescents (*n* = 712; private schools *n* = 384 and public schools *n* = 328) who were present in school on the survey day and had provided > 70% of complete information and a written, informed assent to participate in the study comprised the final sample for analysis. The data were analyzed using IBM SPSS for Windows version 20.0 (IBM Corporation, Armonk, NY, USA). Assuming a normal distribution, the demographic variables, eating habits, unhealthy snack consumption patterns, food environments, and perceptions were compared between private and public-school adolescents using frequency analysis, chi-square statistics, cross-tabulation, and student t-tests. For the comparative analysis of perceptions between private and public-school adolescents, the response options of ‘strongly agree’ and ‘agree’ were categorized as a single category and ‘strongly disagree’ and ‘disagree’ as another while the response option ‘neither agree nor disagree’ was retained as a separate category. Univariate regression analyses were performed to determine the unadjusted effects of independent variables – demographic and food environment characteristics, eating habits, and adolescents’ perceptions on the dichotomized dependent variables- unhealthy snack consumption (highest tertile of aggregate scores indicating high consumption of unhealthy snacks and moderate/lowest tertiles of consumption) in separate models for private and public-school adolescents. Next, we used a backward stepwise logistic regression method to eliminate the least significant predictor variables and derive a reduced model that best explained the data. The independent variables that remained were entered as the covariates in the final multivariate regression model and the adjusted odds ratio with 95% confidence interval (CI) of odds ratio were calculated, considering *p* values ≤ 0.05 as a measure of significant association between variables.

## Results

### Demographic variables

Of the 712 adolescents who completed the survey, 48.2% were girls, 46.1% attended public schools and 50.8% were sixth graders (Table 1)**.** The mean age of adolescents was 10.9 (1.1) years. Significant differences were observed in parents’ education status and family’s monthly income between adolescents attending private and public schools.

**Table 1:** Demographic characteristics of 10–12 years old adolescents in the study

Characteristics	Overall	Public Schools	Private Schools	*P*-value
**Number of Participants**	712 (100.0)	328 (46.1)	384 (53.9)	0.532
**Sex**				
Boys	369 (51.8)	192 (58.5)	177 (46.1)	0.267
Girls	343 (48.2)	136 (41.5)	207 (53.9)	
**Grade in which studying**				
6^th^ Grade	362 (50.8)	174 (53.0)	188 (49.0)	0.537
7^th^ Grade	350 (49.2)	154 (47.0)	196 (51.0)	
**Age (**in years) ^a^	10.9 (1.1)	11.2 (1.1)	10.8 (0.9)	
**Religion**				
Hindu	427 (60.0)	214 (65.2)	213 (55.5)	
Muslim	234 (32.9)	105 (32.0)	129 (33.6)	< 0.001^**^
Christian	32 (4.5)	5 (1.5)	27 (7.0)	
Others (Jain, Parsi, Buddhist)	19 (2.7)	4 (1.2)	15 (3.9)	
**Type of living arrangement**				
Nuclear family	418 (58.7)	121 (36.9)	297 (77.3)	0.004^*^
Joint family	189 (27.8)	138 (42.1)	60 (15.3)	
Extended family	96 (13.5)	69 (21.0)	27 (7.0)	
**Education of father**				
Professional	164 (23.0)	50 (15.2)	114 (29.7)	
Postgraduate or graduate	344 (48.3)	120 (36.6)	224 (58.3)	< 0.001^**^
Post high school/ high school	116 (16.3))	78 (23.8)	38 (9.9)	
Middle/ primary school certificate	70 (9.8)	62 (18.9)	8 (2.1)	
Illiterate	18 (2.5)	18 (5.5)	0 (0.0)	
**Education of mother**				
Professional	76 (10.2)	2 (0.6)	74 (19.3)	
Postgraduate or graduate	256 (36.0)	67 (20.4)	189 (49.2)	
Post high school/ high school	200 (28.1)	117 (35.7)	83 (21.6)	< 0.001^**^
Middle/ primary school certificate	118 (16.6)	92 (28.0)	26 (6.8)	
Illiterate	62 (8.7)	50 (15.2)	12 (3.1)	
**Monthly family income (INR**)				
< 30,000	117 (20.6)	114 (41.0)	3 (1.0)	
< 50,000	164 (28.0)	109 (39.2)	55 (17.9)	< 0.001^**^
50,000- 1,00,000	182 (31.2)	54 (19.4)	128 (41.6)	
> 1,00,000	123 (20.2)	1 (0.0)	122 (39.6)	

*INR* Indian Rupee, ^*^p value < 0.05; ^**^p value < 0.001

Data are presented as number (percentage) or.^a^ mean (standard deviation)

*p* values are obtained from chi-square tests to estimate the difference between adolescents attending public and private schools

### Eating habits of adolescents

Breakfast was ‘never’ consumed at home before leaving for school by 28.8% and tiffin (lunch box) was ‘sometimes’ carried to school by 65.4% of adolescents. Overall, 22.3% reported ‘never’ carrying fruits in their tiffins (lunch boxes), 41.2% ‘often’ or ‘frequently’ carried unhealthy snacks such as biscuits, chocolates, or wafers to school and 70.6% mentioned watching television or screen devices while eating meals at home at least 5–6 times/ week (response options, ‘frequently’ or ‘always’) (Table 2).

**Table 2:** Eating habits and school and home food environment characteristics of 10–12 years old adolescents (*n* = 712) in the study

Characteristics	Never	Sometimes	Often	Frequently	Always
**Eating habits **^**a**^					
Have breakfast at home before leaving for school	205 (28.8)	188 (26.4)	115 (16.2)	110 (15.4)	94 (13.2)
Carry Snacks (Tiffin) to School	32 (4.5)	365 (51.3)	119 (16.7)	132 (18.5)	64 (9.0)
Carry fruits to school	159 (22.3)	267 (37.5)	139 (19.5)	108 (15.2)	39 (5.5)
Carry healthy snacks to school	82 (11.5)	210 (29.5)	176 (24.7)	156 (21.9)	88 (12.4)
Carry unhealthy snacks to school	55 (7.7)	111 (15.6)	212 (29.8)	293 (41.2)	41 (5.8)
Watch television/ screens while eating at home	30 (4.2)	71 (10.0)	108 (15.2)	312 (43.8)	191 (26.8)
**School food environment **^**b**^					
Frequency of purchasing any food/beverage at school	79 (11.1)	91 (12.8)	312 (43.8)	118 (16.6)	112 (15.7)
Frequency of availability of specific foods at school					
a.Fruits, fruit juices	326 (45.8)	156 (21.9)	129 (18.1)	**-**	101 (14.2)
b.Healthy Snacks (poha, upma, sandwich, roti /rice)	289 (40.6)	187 (26.3)	134 (18.8)	**-**	102 (14.3)
c.Unhealthy Snacks (samosa/ vada pav/ pav bhaji)	19 (2.7)	45 (6.3)	36 (5.1)	**-**	612 (86.0)
Frequency of purchasing specific foods at school					
a.Fruits, fruit juices	331 (46.5)	212 (29.8)	141 (19.8)	**-**	28 (3.9)
b.Healthy Snacks (poha/upma/ sandwich/ roti/rice)	286 (40.2)	211 (29.6)	171 (24.0)	**-**	44 (6.2)
c.Unhealthy Snacks (samosa/ vada pav/ pav bhaji/)	204 (28.7)	188 (26.4)	172 (24.2)	**-**	148 (20.8)
**Home Food Environment **^**a**^					
**Availability of foods at home**					
a.Fruits	110 (15.4)	94 (13.2)	101 (14.2)	254 (35.7)	153 (21.5)
b.Vegetables	5 (0.7)	22 (3.1)	55 (7.7)	334 (46.9)	296 (41.6)
c.Healthy snacks	168 (23.6)	155 (21.8)	132 (18.5)	176 (24.7)	81 (11.4)
d. Unhealthy snacks	19 (2.7)	58 (8.1)	102 (14.3)	221 (31.0)	312 (43.8)
a.Carbonated beverages	202 (28.4)	191 (26.8)	145 (20.4)	110 (15.4)	64 (9.0)
**Accessibility and Visibility of foods at home**					
a.Fruits	222 (31.2)	185 (26.0)	113 (15.9)	109 (15.3)	83 (11.7)
b.Healthy snacks	166 (23.3)	153 (21.5)	138 (19.4)	172 (24.2)	83 (11.7)
c.Unhealthy snacks	23 (3.2)	67 (9.4)	96 (13.5)	228 (32.0)	298 (41.9)
d.Carbonated beverages					
**Family Dietary Habits**					
a.Have evening meals together	99 (13.9)	89 (12.5)	115 (16.2)	91 (12.8)	318 (44.7)
b.Have at least a meal at the dining table	153 (21.5)	167 (23.5)	93 (13.1)	129 (18.1)	170 (23.9)
c.Eats out at restaurants/ orders takeaways	213 (29.9)	343 (48.2)	92 (12.9)	52 (7.3)	12 (1.7)

^a^ Response Options- Never (0 days), Sometimes (1–2 days), Often (3–4 days), Frequently (5–6 days), Always (6–7 days)

^b^ Response- Never (0 days), Sometimes (1–2 days), Often (3–4 days), Almost always (5–6 days); Data is presented as number (percentage)

### School and home food environments

Overall, 76.1% reported purchasing foods and/or beverages at schools at least 1–2 times a week, 86.0% mentioned that unhealthy snacks were ‘almost always’ available for sale at canteens and 45.0% had purchased unhealthy snacks at school > 3 times in the past week. The predominant factors determining the purchase of specific foods at school were reported as taste (70.9%) followed by price (48.0%) and availability of foods (32.2%) at schools. Only 44.7% had evening meals together with their family ‘every day’, 23.5% would ‘sometimes’ have meals at the dinner table and 70.1% had eaten out at restaurants/ordered takeaways at least 1–2 times in the last week. While 44.8% reported healthy snacks to be ‘never/ sometimes’ kept in easy to reach places at homes, 73.9% mentioned that snacks such as wafers, *namkeen (ready to eat savory snacks)*, biscuits, *Kurkure* (Indian fried corn puffs) were ‘always/frequently’ kept in easy to reach places (Table 2).

### Frequency of consumption of unhealthy snacks

The weekly consumption frequencies of unhealthy snack items are presented in Table 3. The most frequently consumed unhealthy snacks were biscuits/ cookies (5.2d/wk) followed by wafers/ potato chips (3.4d/wk) and Indian fried snacks- *samosa/ vada pav* (2.8 d/wk). Private school participants reported a higher frequency of consumption of fast foods such as *Pav bhaji* (Indian dish consisting of soft breads and mashed vegetable curry) and burgers/ pizzas, snacks with added salt (wafers/ potato chips and fried rice/ noodles), and carbonated beverages than those from public schools. The frequencies of consumption of relatively lower-priced snacks such as biscuits/ cookies and *Chinese bhel *(popular street food in Mumbai) were observed to be more among public school participants. No significant differences were observed in the frequency of consumption of *samosa/ vada pav*, instant noodles, and chocolates between public and private school adolescents.

**Table 3:** Weekly consumption (mean number of days/ week) of unhealthy snacks in 10–12 years old adolescents in Mumbai, India

Unhealthy snack items	Weekly Consumption (days/ week) †					
	**Overall (*n* = 712)**		**Public school (*n* = 328)**		**Private school (*n* = 384)**	
	Mean	95% CI	Mean	95% CI	Mean	95% CI
**High in fat/fast foods**						
Pav bhaji (butter toasted soft breads with mashed vegetable curry)	1.3	1.1 – 1.6	0.8	0.5 – 1.1	1.6 ^**^	1.4- 1.8
Burger/Pizza	0.6	0.4—0.8	0.2	0.1 – 0.3	1.1 ^**^	0.9 – 1.3
Samosa/vada pav	2.8	2.4- 3.2	3.2	3.0 – 3.4	2.9	2.7 – 3.0
Instant noodles	2.1	1.9—2.3	1.9	1.7 – 2.1	2.2	1.9 – 2.4
**Snacks with added sugar**						
Biscuits/cookies	5.2	5.0 – 5.4	6.9	6.6 – 7.2	4.5 ^**^	4.2 – 4.7
Cakes/Pastries	1.0	0.9—1.1	0.7	0.5 -0.9	1.4 ^**^	1.2 – 1.6
Chocolates	3.9	3.6 – 4.2	3.8	3.4 – 4.3	3.9	3.5- 4.3
**Snacks with added salt**						
Wafers/Potato chips	3.4	3.1—3.7	2.9	2.7 – 3.1	3.7 ^*^	3.5 – 3.9
Fried rice/ noodles	1.2	1.0 – 1.5	0.8	0.6 – 1.1	1.6 ^**^	1.2—2.0
Frankie (potato-filled refined flour wraps)	1.9	1.7 – 2.1	1.6	1.4 – 1.8	2.2	2.0 – 2.4
Chinese bhel (puffed rice tossed with chili sauce, soy sauce, chopped onion, and tomato)	1.2	1.0 – 1.4	1.5	1.2 – 1.8	0.8 ^**^	0.6 – 1.0
**Carbonated beverages**	0.9	0.6 – 1.2	0.3	0.1 -0.5	1.7 ^**^	1.5 – 1.9

^*^p-value ≤ 0.05, ^**^p-value < 0.001

^**†**^ Weekly consumption values are presented as mean and 95% Confidence Interval (CI)

### Comparison of eating habits, unhealthy snack consumption, and food environments between public and private school adolescents

As shown in Supplementary Table 2, a higher frequency of carrying snacks (tiffin), fruits, and healthy snacks to school was observed in private school adolescents as compared to those attending public schools. However, there were no significant differences in the weekly frequency of carrying unhealthy snacks to school or watching television/screens while eating**.** Private school adolescents reported a higher frequency of consuming snacks high in fat/ fast foods and carbonated beverages but not snacks with added sugar or salt. Regarding the characteristics of food environments at schools and home, differences were observed in the availability of healthy snacks at schools, availability of fruits and carbonated beverages at home, frequency of having evening meals together, and perceived parental control during mealtime scores in private and public-school adolescents.

### Comparison of perceptions related to eating behaviors and food environment between public and private school adolescents

More than two thirds (67.1%) mentioned that ‘fruits are not available at school canteens’, three fourths (76.1%) reported that ‘home-cooked foods are always same and boring’ and the majority (87.8%) agreed that the ‘snacks brought from home become cold and unpleasant’ (Table 4). A higher proportion of private school participants agreed to have enough knowledge to make healthy food choices (self-efficacy) and try to eat fruit every day (readiness to change) as compared to those from public schools (*p* < 0.05).

**Table 4:** Perceptions related to food environments in 10–12 years old adolescents in Mumbai (n (%) reporting strongly agree/agree^#^)

**Perceptions**	**Overall** **(*n* = 712)**	**Public School (*n* = 328)**	**Private School (*n* = 384)**	χ^2^	***P* value**
**Perceived Susceptibility & Severity**					
Unhealthy eating habits can increase the risk of health problems	432 (60.7)	172 (52.4)	260 (67.7)	17.324	< 0.001^**^
I can become sick if I don’t eat enough fruits and vegetables	318 (44.7)	134 (40.9)	184 (47.9)	3.521	0.063
Children cannot get diabetes or have high blood pressure	308 (43.3)	118 (36.0)	190 (49.5)	13.115	0.003^*^
**Perceived Benefits**					
Fruits can fight infections	567 (79.6)	272 (82.9)	295 (76.8)	4.049	0.054
Vegetables are good for the eyes, bones, and brain	512 (71.9)	276 (84.1)	236 (61.5)	44.669	< 0.001^**^
Eating breakfast every day will help me to study better in school	458 (64.3)	209 (63.7)	249 (64.8)	0.093	0.760
Eating less unhealthy snacks will keep me fit and strong	587 (82.4)	288 (87.8)	299 (77.9)	11.971	< 0.001^**^
**Perceived Barriers**					
Snacks brought from home become cold and unpleasant	625 (87.8)	285 (86.9)	340 (88.5)	0.421	0.516
Fruits are not available at the school canteen	478 (67.1)	238 (72.6)	240 (62.5)	8.169	0.004^*^
Healthy food items (eg fruits/milkshakes) sold in school are expensive	359 (50.4)	189 (57.6)	170 (44.3)	12.500	0.003^*^
The homecooked foods are always the same and boring	542 (76.1)	243 (74.1)	299 (77.9)	1.414	0.236
Salads are rarely served with meals at home	390 (54.8)	219 (66.8)	171 (44.5)	35.462	< 0.001^**^
**Readiness to Change**					
I try to eat fruits in between my meals every day	418 (58.7)	162 (49.4)	256 (66.7)	2.624	0.005^*^
I want to improve my snacking habits	289 (40.6)	119 (36.3)	170 (44.3)	4.687	0.031^*^
**Self -efficacy**					
I have enough knowledge to make healthy food choices	456 (64.0)	196 (59.8)	260 (67.7)	4.789	0.028^*^
I can prepare a snack or have fruit as a snack at home	301 (42.3)	132 (40.2)	169 (44.0)	1.045	0.306

^#^Values are expressed as number with percentages in parentheses; ^*^p-value ≤ 0.05, ^**^p-value < 0.001

^#^ Perceptions are based on the Health Belief Model measured on a 3-point scale- Strongly disagree/disagree, neutral, agree/strongly agree

Comparison of data was done using Pearson’s χ.^2^ test of independence, df = 1

### Factors associated with unhealthy snacks consumption

Among the adolescents attending public schools, the odds of unhealthy snack consumption were 1.75 times higher in boys, 2.60 times higher when unhealthy snacks were available > 3 days/ week at schools, and 0.89 times lower when meals were had at the dinner table more frequently (Table 5). Also, higher consumption of unhealthy snacks was observed to be associated with greater perceived barriers in school and home food environments and lower readiness to change eating habit scores. For the private school attending adolescents, we observed that mothers’ education (OR 0.78, 95% CI 0.66–0.82, < 0.001), family income (OR 2.15, 95% CI 1.98–2.32, *p* < 0.001), availability of unhealthy snacks (OR 2.98, 95% CI 1.36–3.46, *p* < 0.001) and fruits at home (OR 0.57, 95% CI 0.49–0.69, *p* < 0.001), having evening meals together (OR 0.71, 95% CI 0.63–0.81, p 0.031), and perceived parental control during mealtimes ( OR 0.67, 95% CI 0.62–0.72, *p* < 0.001) were associated with consumption of unhealthy snacks.

**Table 5:** Regression analyses to determine factors associated with unhealthy snack consumption^a^in adolescents in Mumbai

**Variables**	Public School Adolescents (*n*= 328)			Private School Adolescents (*n*= 384)		
	**OR**	**95% CI**	***p*-value**	**OR**	**95% CI**	***p*-value**
**Socio-demographic characteristics**						
Sex- Boys (Ref: Girls)	**1.75**	**0.69 - 0.79**	0.032^*^	1.13	0.93 - 1.22	0.381
Education of father ( Ref: < High school certificate)	1.05	0.98 - 1.12	0.132	1.83	1.78 - 2.02	0.221
Education of mother (Ref: < High school certificate)	1.09	0.91 - 1.27	0.191	0.78	**0.66 - 0.82**	<0.001^**^
Monthly family income (Ref: < 30000 INR)	1.03	0.92 - 1.10	0.619	2.15	**1.98 - 2.32**	<0.001^**^
**Eating Habits**						
Breakfast before going to school (Ref: <3 d/ week)	0.99	0.95 - 1.06	0.672	** 0.77**	**0.71 - 0.83**	<0.001^**^
Carry Snacks (Tiffin) to School (Ref: <3 d/ week)	**0.83**	**0.81 - 0.86**	0.028^*^	1.06	0.98 - 1.14	0.563
**School Food Environment**						
Availability of fruits (Ref: <3 d/ week)	1.01	0.88 - 1.12	0.421	1.01	0.98 - 1.10	0.066
Availability of healthy snacks (Ref: <3 d/ week)	1.02	0.99 - 1.06	0.203	0.99	0.82 - 1.26	0.083
Availability of unhealthy snacks (Ref: <3 d/ week)	**2.60**	**1.44 - 3.79**	<0.001^**^	0.99	0.93 - 1.08	0.328
Reasons for purchasing snacks – Taste (Ref: No)	0.99	0.95 - 1.04	0.198	** 2.87**	**2.36 - 3.03**	< 0.001^**^
Reasons for purchasing snacks – Price (Ref: No)	1.06	1.01 - 1.10	0.319	** 1.65**	**1.44 - 1.83**	< 0.001^**^
**Home Food Environment **						
Availability of fruits at home (Ref: <3 d/ week)	0.99	0.98 - 1.07	0.921	** 0.57**	**0.49 - 0.69**	< 0.001^**^
Availability of unhealthy snacks at home (Ref: <3 d/ week)	1.23	0.98 - 1.41	0.332	**2.98**	**1.36 - 3.46**	< 0.001^**^
Visibility of fruits in easy to reach places (Ref: <3 d/ week)	1.08	0.95 - 1.16	0.712	0.88	0.72 - 1.01	0.089
Visibility of unhealthy snacks in easy to reach places (Ref: <3 d/ week)	1.07	0.96 - 1.18	0.441	1.17	0.98 - 1.28	0.331
At least a meal at the dining table (Ref: <3 d/ week)	**0.89**	**0.76 - 0.95**	0.023^*^	1.06	1.01 - 1.12	0.448
Evening meals together (Ref: <3 d/ week)	1.02	0.94 - 1.13	0.782	**0.71**	**0.63 - 0.81**	0.031^*^
Eating out in restaurants (Ref: <3 d/ week)	0.99	0.95 - 1.04	0.112	0.94	0.83 - 1.28	0.237
**Perceived parental control at mealtimes **(Ref: strongly disagree, neutral, and disagree)						
Pressure to eat veggies during meals	1.03	0.99 - 1.06	0.313	1.12	1.04 - 1.20	0.444
Parents choose time to have snacks	1.14	0.98 - 1.28	0.271	**1**.01	0.97 - 1.15	0.521
Allowed to eat fast foods only on weekends	1.01	0.93 -1.04	0.289	**0.67**	**0.62 - 0.72**	< 0.001^**^
**Adolescents’ Perceptions (**Ref: strongly disagree, neither agree nor disagree and disagree)						
Perceived susceptibility (Unhealthy eating habits can increase risk of health problems)	1.01	0.92 - 1.12	0.662	1.01	0.94 - 1.18	0.911
Perceived benefits (Fruits can fight infections)	0.99	0.98 - 1.08	0.344	1.16	0.97- 1.22	0.652
Perceived benefits (Vegetables are good for…)	0.91	0.88 - 1.12	0.218	0.99	0.89 - 1.19	0.0871
Perceived barriers (Fruits are not available at school)	**1.98**	**1.67- 2.31**	0.003^*^	**2.03**	**1.96 - 2.12**	< 0.001^**^
Perceived barriers (Homecooked foods are the same….)	1.11	0.97 - 1.22	0.066	**3.11**	**2.34 - 4.65**	< 0.001^**^
Perceived barriers (Healthy foods are expensive)	**4.97**	**2.98 - 5.19**	<0.001^**^	**1.78**	**1.32 - 2.22**	< 0.001^**^
Readiness to change (I try to eat fruits every day)	** 0.79**	**0.62 - 0.92**	0.009^*^	1.11	0.99 - 1.24	0.512
Self–efficacy (I can prepare a snack or have fruit….)	0.99	0.93 - 1.08	0.123	**0.88**	**0.76 - 0.99**	0.043^*^

*OR* odds ratio, *CI* confidence interval. * *p* ≤ 0.05, **p < 0.001

^a^ Dependent variable- Highest tertile of unhealthy snack consumption compared with the low and moderate tertiles of consumption of unhealthy snacks and carbonated beverages/d in adolescents

OR values that show significant associations with higher consumption of unhealthy snacks are highlighted

## Discussion

This study is one of the few to concurrently explore the characteristics of school and home food environments of adolescents in India and the first to examine the socioeconomic, food environmental, familial, and intrapersonal factors of unhealthy snack consumption in Indian adolescents. The results highlighted that the correlates of unhealthy snack choices in adolescents are multifaceted and range from demographic characteristics to various social and food environmental factors within the adolescents’ immediate settings at schools and homes. The key findings that emerged from this study were 1) a frequent consumption of unhealthy snacks high in fats, added sugar, and salt among adolescents, irrespective of the type of school attended (used as a proxy indicator for SES), 2) a pervasiveness of energy-dense and nutrient-poor snacks in the school and home food environments of adolescents, 3) low levels of perceived severity of adverse consequences of unhealthy snack consumption, readiness to change habits and perceived parental control during mealtimes, and 4) a significant association of maternal education level, family income, availability of unhealthy snacks at schools and homes, frequency of having family meals and adolescents’ own perceived barriers within the food environments with their unhealthy snack consumption patterns.

In general, the adolescents attending private schools reported healthier eating habits (such as higher frequency of having breakfast at home before going to school, carrying fruits as snacks to school, and purchasing healthy snacks at school) and food environments (such as greater availability of fruits for sale at schools, better accessibility of fruits and healthy snacks at homes, and a higher perceived parental control during mealtime) than those attending public schools. These findings can be explained by a higher SES of private school families that might have resulted in better affordability of relatively higher-priced fruits and healthy snacks [26, 50] and a higher level of parents’ education that could have contributed to greater awareness of the benefits of healthy eating and more supportive family food environments [25]. Previous studies, conducted outside India have reported similar associations between low socioeconomic position, low availability of healthy foods at home, poor mealtime habits, and limited parental encouragement of healthy eating habits in adolescents [51–53].

In contrast to the majority of evidence from high-income countries that suggest higher consumption of cheap, energy-dense unhealthy foods among lower SES [50, 53, 54], we observed a somewhat blurring line in the SES-related differentials in adolescents’ unhealthy snack consumption in the present study. While the private-school adolescents reported more frequent consumption of expensive unhealthy snacks such as restaurant bought burgers and pizzas or branded processed foods with added sugar and salt such as cakes and wafers, a higher frequency of consumption of the cheaper versions of similar nutritionally poor, high-calorie snacks such as biscuits, fried Indian snacks, and unhealthy street foods was observed in adolescents attending public schools. In the context of the ongoing nutrition transition and its impact on food preferences and health outcomes in adolescents, these findings draw concerns regarding frequent consumption of unhealthy snacks in urban adolescents in India, irrespective of their socioeconomic backgrounds. Given the well-documented associations between unhealthy food consumption and risk of nutrition-related lifestyle disorders [55, 56], age and culturally appropriate initiatives that build awareness and foster positive attitudes are increasingly warranted to limit unhealthy snacking habits in Indian adolescents across SES.

Adolescents spend a substantial amount of time at schools, so health-promoting school food environments can act as enablers of reduced unhealthy snack consumption. In our study, the majority of participants reported purchasing foods and/ beverages at school canteens. The most frequently bought foods at school canteens, irrespective of the type of school attended, were unhealthy snacks such as wafers, chocolates, and fried Indian snacks- *samosa and vada pav*. While fruits and healthy snacks were reported to be available in the private school canteens, these items were rarely bought as snacks. This suggests that the availability of fruits and healthy snacks are necessary to allow access to healthy food options but may not necessarily result in better intake unless the availability of unhealthy foods for sale in schools is restricted. Similar insights were reported in previous studies [37, 57–60]. Thus, suitable measures must be taken to simultaneously reduce access to unhealthy foods and improve the provision of healthy food and beverage alternatives to ‘tasty’ and ‘cheap’ unhealthy snacks at schools in India. Introducing behavior change interventions to improve acceptability and sustainability of modifications within school food environments and employing stringent school food policies regarding accessibility to nutrient-poor and energy-dense snacks at school canteens may add further leverage.

Besides the influences of school food environments, analyses showed associations of familial factors such as frequency of eating out, having dinner at the table, and having evening meals together and home food environment characteristics such as availability and accessibility of unhealthy snacks at home with the consumption patterns. Better availability of fruits at home, having meals at the dinner table and greater parental control during meals were observed to be associated with lower unhealthy snack consumption. These results are consistent with previous studies [22, 61–64], and reiterate the significance of modifying the key dimensions of food environments at homes such as encouraging family mealtimes and increasing the availability of fruits and healthy snacks at home to improve eating behaviors in adolescents. Although this study did not assess parental intakes or perceptions, our findings indicated that parents’ encouragement to adolescents to include vegetables at meals, setting family rules, and improving home availability of healthy foods can result in a lower frequency of consumption of unhealthy snacks. This brings forth the critical roles that parents play in the pursuit of improving the overall diet quality of adolescents. Targeted strategies that build nutritional knowledge and instill positive attitudes towards the benefits of healthy eating among parents along with a supportive home food environment are likely to encourage healthier food choices in adolescents.

As per the Health Belief Model, the perceived benefits of adopting desired behaviors refer to an individual’s perception of the value or importance of engaging in a health-promoting behavior. Self-efficacy, on the other hand, is a function of an individual’s belief that he/ she has the necessary skills and resources to overcome the barriers and the ability to perform and execute behaviors that are necessary to bring desired behavior changes. The finding that public school students had a greater perceived benefit and lower self-efficacy score indicates that they appreciated and acknowledged the benefits of healthy eating habits but had limited confidence and efficacy to take action. This can be explained by the public-school adolescents reporting higher perceived barriers within their food environments as compared to their private school counterparts, which might have contributed to lower self-efficacy scores. These results highlight the importance of not only building knowledge and awareness regarding healthy eating habits but also for ensuring that the adolescents develop the necessary skills required to translate the knowledge gains into healthier snacking habits and better dietary practices.

Another significant observation was the influence of perceptions of the barriers and facilitators within the food environments on adolescents’ snack consumption. In this study, negative perceptions (limited availability of fruits at school, relatively high cost of healthy snacks, and lack of variety in home-cooked foods) and positive attitudes (readiness to change and self-efficacy when adopting healthy habits) were associated with the frequency of unhealthy snack consumption in adolescents. These findings corroborate the evidence from previous qualitative investigations [31, 34] and quantitative studies grounded in health behavior theories [5, 31, 48, 65]. Integrating food environment interventions with policy approaches that address perceived barriers and endorse social support through active peer and family engagement may facilitate and maintain reduced unhealthy snack consumption. Results pertaining to the differences in the perceptions of private and public-school adolescents, particularly for the perceived barriers of food environments imply that socio-economic disparities in the perceptions and the related opportunities and challenges to intervening within the food environments must also be considered in future advocacy efforts.

There are a few limitations of this study. First, adolescents, ages 10–12 years were selected for the study. This sample selection method limits the generalizability of the results to younger or older adolescents in India. Yet, the period of early adolescence is considered an important phase of life when individuals start to develop an understanding of how their present eating habits may influence future wellbeing. Investigating the factors that influence unhealthy snacking behaviors at this age is likely to pave way for early intervention strategies with long-lasting health benefits. Similar studies must consider selection of more schools for greater representation, and inclusion of adolescents across age categories to improve the generalizability of the findings. Also, we investigated the socioeconomic differences in variables using the type of schools attended by adolescents as a proxy measure of SES. Assessment of a broader set of SES related variables in future studies may further establish the socioeconomic correlates of unhealthy snacking patterns among adolescents. Second, the measures were self-reported, so they can be subject to recall or reporting bias. However, to minimize the bias, we evaluated the psychometric properties of the questionnaire used to capture food environmental factors before administration in adolescents and used a validated food frequency questionnaire to estimate unhealthy snack consumption. Future research can employ methods for direct observations of availability, price, and quality of foods sold in schools and of factors within a home food environment such as the availability and visibility of foods by trained raters to reduce any potential bias in self-reported observations. Third, given the complex and multifactorial impact of social and food environments on eating behaviors, some factors could have been missed, though attempts were made to include most of the common and relevant determinants, as indicated in previous studies.

The novelty of this study lies in the evaluation of different individual and environmental factors in a single study and an investigation into the influences of these exposures on unhealthy snack consumption in adolescents. Several food-environmental and individual-level factors were identified as correlates of unhealthy snack consumption in adolescents, presenting targets for future intervention programs. Given that the access and affordability of foods can influence the snacking behaviors of adolescents and that the SES differentials in food environments of adolescents in India are poorly understood, we compared the determinants of unhealthy snack consumption between adolescents attending private and public schools. The results suggested that socio-demographic characteristics such as gender, maternal education, and SES can be used to customize interventions and that a focus on the role of parents in shaping adolescents’ diet quality must be emphasized. For low SES families, the findings reiterated the need for initiatives that improve the affordability of relatively higher-priced healthier fruits and vegetables and foster greater nutrition-related awareness whilst ensuring a reduced availability of cheap, nutritionally poor snacks such as biscuits, fried Indian snacks, and unhealthy street foods in school and home food environments. Moreover, the results showed that the private school canteens and higher SES households had better availability of fruits and healthy snacks but also had a wider range of unhealthy snack options, easily accessible to adolescents. This suggests that higher SES families must be motivated to restrict the availability and accessibility of unhealthy snacks at schools and homes and be encouraged to continue maintaining a greater availability of healthy snack options and leveraging the family mealtimes to improve the consumption of fruits and vegetables.

Other strengths of this study were that a wide range of perception related variables such as perceived susceptibility and severity of adverse consequences of unhealthy eating behaviors, perceived barriers within food environments, and readiness to change and self–efficacy to adopt healthier dietary practices along with the personal factors that guide the food purchase decisions such as taste and convenience were investigated as correlates of unhealthy snacking habits. Results provided valuable insights into the associations between adolescents’ eating habits, parental control during mealtimes, and adolescents’ unhealthy snack consumption. The findings of this study attempt to draw attention to the frequent availability of low-cost unhealthy snacks at schools, and limited availability of healthy snack options at home, and a need to integrate positive perceptions of healthy eating habits and self-efficacy skills into adolescent health and nutrition policies.

## Conclusions

This study presented a comprehensive assessment of school and home food environments, and perceptions of eating behaviors in adolescents and how they come together to moderate adolescents’ unhealthy snack consumption. The paucity of food environment research in India underpins the need to conduct a situational analysis of the local, neighborhood, school, and home food environments of adolescents. Additional research is also required to determine the socio-cultural, environmental, and contextual correlates of food choices in a larger and diverse sample. The findings posit that exploring the determinants of food choices within the immediate food environments of adolescents and designing feasible interventions targeted at optimizing these exposures present critical avenues to limit unhealthy snacking behaviors in adolescents. Since food environmental factors consistently interact with personal perceptions, attitudes, and motivations to engage in healthy eating behaviors, addressing the interplay of these factors may confer greater benefits on present and future health outcomes in adolescents.

## Supplementary Information

**Additional file 1: Supplementary Table 1.**Summary of measures included in the questionnaire assessing eating habits, consumption patterns, food environments and perceptions of 10-12 years old adolescents (*n*=712) in Mumbai, India 

**Additional file 2:** **Supplementary Table 2. **Comparison of mean eating habit, unhealthy snack consumption and food environment characteristic scores between adolescents attending public and private schools

## Data Availability

All data generated and /or analyzed during the current study are included in the published article and its supplementary information files.
