# Supplementary material for: Socioeconomic, intrapersonal and food environmental correlates of unhealthy snack consumption in school-going adolescents in Mumbai
Source: BMC Public Health. 2022 Jun 6;22:1129. doi: 10.1186/s12889-022-13449-6 (PMC9171983; doi:10.1186/s12889-022-13449-6)
Supplement: Supplementary file 1 — Additional file 1: Supplementary Table 1.Summary of measures included in the questionnaire assessing eating habits, consumption patterns, food environments and perceptions of 10-12 years old adolescents (n=712) in Mumbai, India [file 12889_2022_13449_MOESM1_ESM.docx]

**Supplementary Table 1**: Summary of measures included in the questionnaire assessing eating habits, consumption patterns, food environments and perceptions of 10-12 years old adolescents (n=712) in Mumbai, India

| **Measures** | **Description** | **Example Questions** | **Response Categories** | **Response Scoring** |
| --- | --- | --- | --- | --- |
| **Sociodemographic Characteristics** | **Adolescent reported items**-age, gender, class in which studying, religion  **Parent-reported** **items**: Parents’ highest education, and monthly family income | Please specify…. | Depending on the question | - |
| **Eating Habits** | **Weekly frequency** of consuming breakfast, carrying lunch (*tiffin)* to schools, viewing television/screens while eating | In the last 7 days, how often did you perform the following…? | *Never* (0 days)  S*ometimes* (1-2 days)  *Often* (3-4 days)  *Frequently* (5-6 days)  *Always* (7 days) | 0 to 4 |
| **Consumption of Unhealthy Snacks**  **and Carbonated Beverages** | **Unhealthy snacks (11 items)-** Biscuits, cakes/pastries, chocolates, wafers/potato chips, Indian fried snacks such as samosa, vada pav, pav bhaji, Fried rice/noodles, frankie (potato filled refined flour wraps), burger/pizza, instant noodles. **Carbonated beverages (1 item)** | In the last 7 days, how many days did you consume the following…? | *Never* (0 days)  S*ometimes* (1-2 days)  *Often* (3-4 days)  *Frequently* (5-6 days)  *Always (7 days)* | 0 to 4 |
| **School Food Environment** | **Frequency** of purchasing foods/beverages at school  Type of foods a**vailable for sale** at schools  (fruit items (fresh fruits, 100%fruit juice, fruit milk shakes), healthy snacks (sandwich/poha, upma/ idli/ roti/paratha, unhealthy snacks (fried snacks, wafers, chocolates, noodles, pav bhaji).  Type of foods **frequently purchased** at school- same options as provided above  **Reasons** for purchasing these food items  **(**taste**,** price, availability, and convenience) | In the last month, how often did you buy any food or drink from the school canteen?  How often are the following foods and drinks available at school to buy…?    In the last 7 days, how often did you buy the following foods at school…?  Why do you buy these foods…? | *Never* (< once/month)  S*ometimes* (2-3 times/ month)  *Often* (1-2 times/ wk)  *Frequently* (3-4 times/wk)  *Always* (5-6 times/wk)  *Never* (0 days)  S*ometimes* (1-2 days)  *Often* (3-4 days)  *Almost Always* (5-6 days)  *Never* (0 days)  S*ometimes* (1-2 days)  *Often* (3-4 days)  *Almost Always* (5-6 days)  *4 Reasons, each answered as Yes (1)/No (0)* | 0-4  0-3  0-3    0-4 |
| **Home Food Environment** | **Availability** of fruits, healthy snacks, unhealthy snacks, and sugar-sweetened beverages at home  **Visibility** of fruits, healthy snacks, unhealthy snacks, and sugar-sweetened beverages at easy to reach places at home  **Family dietary habits**- Frequency of eating out in restaurants/ordering takeaways, having family meals, and having meals at the dinner table  **Perceived parental control** at mealtime | In the last 7 days, how often were the following foods available at home/ kept in easy to reach places….?  In the last 7 days, how often did you perform the following…?  Statements (*n* = 3) | *Never* (0 days)  S*ometimes* (1-2 days)  *Often* (3-4 days)  *Frequently* (5-6 days)  *Always* (7 days)  *Same as above*  *Strongly disagree to strongly agree* | 0-4  0-4  0-4 |
| **Perceptions** | **Perceived** susceptibility & severity, benefits, barriers, readiness to change, self-efficacy | Statements based on the constructs of the Health Belief Model (*n* = 16) | *Strongly disagree, disagree, neither agree nor disagree, agree, strongly agree* | 0-4 |
|  |  |  |  |  |
