# Supplementary material for: Socioeconomic, intrapersonal and food environmental correlates of unhealthy snack consumption in school-going adolescents in Mumbai
Source: BMC Public Health. 2022 Jun 6;22:1129. doi: 10.1186/s12889-022-13449-6 (PMC9171983; doi:10.1186/s12889-022-13449-6)
Supplement: Supplementary file 2 — Additional file 2: Supplementary Table 2. Comparison of mean eating habit, unhealthy snack consumption and food environment characteristic scores between adolescents attending public and private schools [file 12889_2022_13449_MOESM2_ESM.docx]

**Supplementary Table 2:** Comparison of mean eating habit, unhealthy snack consumption and food environment characteristic scores between adolescents attending public and private schools

| **Characteristics** | **Range of Scores** | **Overall**  **(n = 712)** | **Public Schools (n =328)** | **Private Schools**  **(n = 384)** | **p values**  0.019^*^  <0.001^**^  <0.001^**^  <0.001^**^  0.067  0.144  <0.001^**^  0.512  0.323  < 0.001^**^ |
| --- | --- | --- | --- | --- | --- |
| **Eating habits** †  Breakfast before school  Carry Snacks (*Tiffin*) to School  Frequency of carrying fruits, healthy and unhealthy snacks to schools   1. Fruits 2. Healthy Snacks (*poha, upma, sandwich, roti/ parantha/rice*) 3. Unhealthy Snacks (*wafers, chocolates, biscuits, noodles*)   Watching television/ screens while eating at home  **Frequency of consuming unhealthy snacks** ‡   1. Snacks high in fat/ fast foods (4 items, each scored 0 to 4) 2. Snacks with added sugar (3 items, each scored 0 to 4) 3. Snacks with added salt (4 items, each scored 0 to 4) 4. Carbonated beverages (1 item, scored 0 to 4) | 0-4  0-4  0-4  0-4  0-4  0-4  0-16  0-12  0-16  0-4 | **Mean (SD)**  2.4 (1.3)  2.7 (1.2)  1.4 (0.2)  2.5 (0.3)  2.8 (0.3)  3.6 (0.8)  13.3 (4.7)  10.8 (1.3)  12.7 (5.1)  2.2 (1.5) | **Mean (SD)**  1.8 (1.1)  2.4 (1.8)  1.3 (0.2)  2.2 (0.3)  2.7 (0.6)  3.7 (1.1)  11.5 (4.2)  10.9 (1.9)  12.3 (4.2)  1.3 (1.1) | **Mean (SD)**  2.9 (1.4)  3.0 (1.5)  1.6 (0.3)  2.7 (0.1)  2.8 (0.7)  3.6 (0.5)  15.7 (3.1)  11.1 (2.4)  13.1 (3.5)  2.8 (1.5) |  |
| ***School Food Environment***  **Frequency of purchasing any food/beverage at school**  **Frequency of purchasing specific foods at school** §   1. Fruits, fruit juices 2. Healthy Snacks (*poha, upma, sandwich, roti/parantha /rice*) 3. Unhealthy Snacks (*samosa, vada pav, pav bhaji, noodles*) | 0-4  0-3  0-3  0-3 | 2.2 (1.2)  0.8 (0.2)  1.1 (0.5)  2.7 (1.4 | 2.3 (1.4)  0.7 (0.3)  0.6 (0.1)  2.8 (1.1) | 2.1 (1.2)  0.8 (0.4)  1.9 (1.0)  2.9 (1.7) | 0.063  0.621  <0.001^**^  0.419 |
| ***Home Food Environment*** †  **Availability of foods at home**   1. Fruits 2. Healthy snacks 3. Unhealthy snacks 4. Carbonated beverages   **Accessibility and Visibility of foods at home**   1. Fruits 2. Healthy snacks 3. Unhealthy snacks 4. Carbonated beverages   **Family Dietary Habits**   1. Have evening meals together. 2. Have meals at the dinner table. 3. Eats out at restaurants/ orders takeaways.   **Perceived parental control during mealtime (3 items on Likert scale)** §§ | 0-4  0-4  0-4  0-4  0-4  0-4  0-4  0-4  0-4  0-4  0-4  0-12 | 3.1 (1.8)  2.8 (1.6)  3.3 (2.0)  2.2 (1.4)  2.2 (1.1)  1.5 (0.7)  1.6 (0.6)  1.8 (1.3)  2.8 (1.1)  3.2 (0.7)  2.1 (1.1)  7.8 (1.3) | 2.9 (2.1)  3.6 (1.1)  2.9 (1.8)  2.8 (2.2)  1.6 (1.0)  1.1 (0.5)  1.6 (0.7)  1.7 (1.1)  2.6 (0.9)  3.3 (0.8)  1.6 (0.8)  6.3 (1.2) | 3.4 (1.6)  3.7 (1.9)  2.8 (1.3)  3.6 (1.8)  2.7 (1.4)  1.8 (1.1)  1.7 (0.8)  1.9 (1.6)  3.1 (0.5)  3.2 (0.7)  2.5 (1.0)  8.6 (1.5) | <0.001^**^  0.450  0.435  <0.001^**^  <0.001^**^  <0.001^**^  0.078  0.056  <0.001^**^  0.075  <0.001^**^  <0.001^**^ |

^*^p-value ≤ 0.05; ^**^ p value < 0.001

†Frequency responses were never (0 d/week) to always (6-7d/week), scored 0 to 4. ‡Frequency responses include never (0 d/week) to 2-3 times a day, scored 0 to 4.

§ Frequency responses were never (0 d/week) to almost always ( 5-6 d/week), scored 0 to 3.

§§ Agreement to each statement was reported on a 5-point Likert scale, response options were ‘strongly disagree’ to ‘strongly agree’, scored from 0 to 4.
